# Ion Pairing and Solvent Shell Polarization in Aqueous Solutions of Divalent Metal Sulfates

**DOI:** 10.1021/acs.jpcb.5c03061

**Published:** 2025-08-06

**Authors:** Erik Bialik, Zareen Abbas

**Affiliations:** Department of Chemistry, 3570 University of Gothenburg Gothenburg SE-413 90, Sweden

## Abstract

The interactions of divalent metal cations with other species in aqueous solution are important in contexts such as the basic functioning of living cells. Recent evidence suggests that contact ion pairs are virtually absent in magnesium sulfate solutions and that solvent-shared ion pairs predominate. It is still unclear whether this is the case for divalent metal salts, in general. The polarization energy of the water molecules of the first solvation shell of divalent metal cations is known to be essential to correctly calculating the ionic solvation energy. Here, we show that the same type of solvent shell polarization is important for ion pairing in metal sulfate model electrolytes. The polarization energy of the solvating water molecules makes them harder to replace with ions compared to nonpolarizable models and therefore suppresses ion contact. As this polarization energy increases strongly with the electric field strength at the position of solvating water molecules, which, in turn, depends on cation size, this introduces an ion size dependence. With a polarizable water model, contact ion pairing is completely suppressed for cations below a certain minimum size. No corresponding tendency is seen with a nonpolarizable water model, for which direct contacts between cations and anions are prevalent for all cation sizes considered. This observation may explain the previously noted tendency of extremely small ions in certain respects to behave as large ions. While this effect has previously been ascribed to a strongly bound solvation shell around small ions, the current results provide a mechanism for why small ions are *disproportionately* strongly solvated.

## Graphical abstract

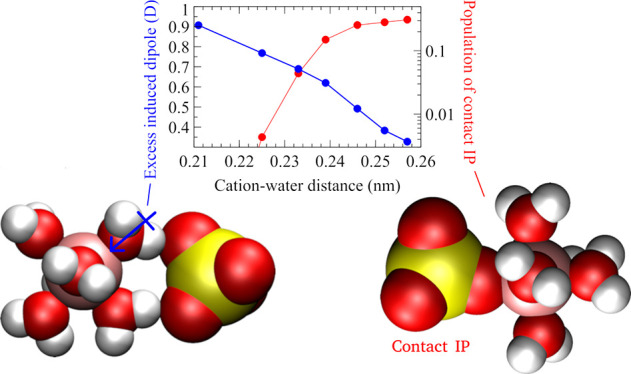

## Introduction

The properties and interactions of metal cations such as Mg^2+^ and Zn^2+^ in aqueous solution are decisive for many important processes, not the least of which are within living cells. Despite the apparent chemical simplicity of aqueous salt solution, the factors that determine the degree and kind of ion pairing that occurs are not completely understood. For instance, the interaction between Mg^2+^ and ATP is of considerable interest as the magnesium ion is necessary for ATP to perform its function as an energy carrier in the cell. Both the CHARMM and AMBER all-atom force fields for protein simulations have recently been found lacking in their ability to describe the Mg^2+^-ATP complex.^1^ There are ongoing efforts to improve force fields,^2–4^ as well as direct experimental investigations of the structure of the complex.^5^ This is only one current problem among many; the state of the art in metal cation force fields as of a few years ago has been extensively reviewed.^6^ A recent benchmark study of force fields for Zn^2+^ found that no known model was generally applicable and recommended that models should be chosen on an ad hoc basis.^7^

Dielectric relaxation spectroscopy, a technique that can distinguish contact ion pairs (CIP), solvent-shared ion pairs (SIP), and solvent-separated ion pairs (2SIP), has been applied to some divalent metal sulfates. An early application to magnesium sulfate solutions, a well-studied test case for ion pairing, reported some population of each of these types of ion pairs, including CIP.^8^ Nickel and cobalt sulfate were found to give similar dielectric spectra.^9^ A recent investigation of zinc sulfate solutions found no 2SIP and a slow water relaxation mode that overlaps with the peak that corresponds to CIP.^10^ A more recent investigation of magnesium sulfate solution with the data interpretation aided by all-atom molecular dynamics (MD) simulations found predominantly SIP, where one water molecule is situated between the anion and cation, and did not give any indication that CIP are present at all.^11^ Terahertz absorption spectroscopy in conjunction with MD simulations gives rise to the same conclusion: that SIP are predominant in magnesium sulfate solutions and that there are no traces of CIP.^12^ The dynamics of water reorientation is considerably slowed in magnesium sulfate solutions for a subset of water molecules, identified as the water molecules situated between magnesium and sulfate ions in SIP.^12–14^

The type and degree of ion pairing are determined by the balance between the ion–ion and ion–water interactions in solution. The solvent-averaged interaction between ions is typically a small (a few *k*_*B*_ *T*) difference between two large (hundreds of *k*_*B*_ *T*) terms that represent the direct ion–ion and ion–solvent interactions. This makes both qualitative and quantitative descriptions of the total thermally averaged interaction difficult. While this is true regardless of the ion charge, the quantitative difficulties are more severe for highly charged ions. The polarizable force field used in ref^12^ and the nonpolarizable one in ref 11 both agree with available experimental evidence, including activity derivatives, and give similar solution structures. In ref 11, the Lennard–Jones (LJ) σ parameter for the Mg^2+^-sulfate O interaction, effectively the cation–anion contact distance, was scaled by a factor of 1.65. This has the effect of suppressing the formation of CIP a priori, as the Mg^2+^ sulfate O distance that corresponds to ‘contact’ falls within the steeply repulsive part of the LJ potential. The fact that such a drastic modification to the interaction potential compared to the polarizable model in ref 12 suggests that polarization suppresses the formation of CIP.

The question of how to treat polarization effects in molecular dynamics simulations remains open. Even in ″nonpolarizable” simulations, the model parameters are chosen to describe the average effect of polarization. In explicitly polarizable simulations, polarizabilities are typically represented by point polarizabilities that are distributed in some way over the molecular species. This increases the complexity of the force field and introduces a failure mode known as the “polarization catastrophe”, where the interaction energy diverges due to the mutual polarization that occurs when polarizable sites come too close to each other.

There has been considerable development in the past decade or so, which points in the direction that polarization effects are essential to capture the energetics of the first solvation shell of divalent metal cations. Notably, considerable effort has been spent to construct accurate models for use within the AMBER and CHARMM force fields. The approaches in these two cases are somewhat different, but they share the physical insight that the polarization of the first hydration shell must be included to allow a reasonable description in terms of both the hydration free energy and the distance to each of the first hydration layer water oxygen atoms. In ref 15, it was shown that the conventional charged LJ-particle model of ions cannot simultaneously reproduce the solvation energy and ion–water distance for divalent metal ions without an additional term to represent the first solvation shell polarization energy. The polarization energy of a point polarizability is given by

$$U^{pol}=\frac{-\alpha }{2}E^{2}$$ 1

where α is the polarizability and *E* ^2^ is the magnitude of the electric field. As both of these quantities are positive, polarization always decreases the total interaction energy. For a single point polarizability interacting with a point charge, the expression becomes

$$U^{pol}=\frac{-\alpha ’q^{2}}{4pi\epsilon_{0}r^{4}}$$ 2

[pi → π], where α′ is the polarizability volume. The set of cation parameters suggested in ref 15 for use with the AMBER protein force field uses a *1*/*r* ^4^ term to take polarization effects into account, in addition to the standard Lennard–Jones terms. In the Drude-polarizable version of the CHARMM force field,^16^ the force-field parameters have been carefully selected to represent the interactions of the divalent magnesium cation.^17^

As the formation of a CIP implies that some number of solvation water molecules have to lose that status to make room for the ion contact, the strong polarization of the cation solvent shell suggests a candidate mechanism for the difference between polarizable and nonpolarizable models. It has previously been reported that ion association in sodium sulfate is suppressed when a polarizable water model is used,^18^ due to the stabilizing effect of polarization on the first solvation shell of the sulfate ion.

## Simulation Methodology

We performed MD simulations for a range of force-field parameters relevant to divalent metal cations to explore the effects of solvent shell polarization on the mode and amount of ion pairing.

For sulfate, we used the nonbonded parameters from ref 11 without the scaling of the cation-sulfate oxygen σ, as well as those from the older parametrization in ref 19, for which the parameter values are significantly different. The former model, we refer to as the “Mamatkulov” model, and the latter as the “Cannon” model of sulfate. The nonbonded parameter sets used here, shown in Table 1, differ only by the O LJ parameters. The ion was kept in a rigid tetrahedral geometry by constraining the O–S distance to 1.52 Å and the O–O distance to 2.49 Å using the SHAKE algorithm.^20^ A Drude shell particle was assigned to the sulfur atom that contained the total sulfur charge, and the spring constant was chosen to give a polarizability volume of 5 Å^3^. This polarizability is smaller than the expected value of about 7 Å^3^,^21^ but larger values resulted in numerical instabilities. The polarizability of the cation was set to zero. We used the polarizable SWM4-NDP water model.^22^

**Table 1:** Force-Field Parameters

particle (pair)	LJ-ó (Å)	LJ-ϵ (kJ/mol)	q (ε_0_)	α′(Å^3^)
SO_4_–S	3.55	1.0465	0	
SO_4_–S (shell)	0.0	0.0	2	5
SO_4_–O (Mamatkulov)	3.916	0.1	–1	
SO_4_–O (Cannon)	3.15	0.8368	–1	
M^2+^(A)	2.6	0.1	2	
M^2+^(B)	3.6	0.1	2	

The modeling choices were made not to make the performance of any model variant quantitatively optimal but to make the difference between the variants with the polarizable and nonpolarizable water models qualitatively interpretable. In particular, the polarizability of the anion has to be treated on the same footing as the polarizability of the water molecules, i.e., using a single central shell particle, so as not to bias the energetics of replacing a water molecule with an anion to form a CIP. To explore the consequences of such bias, we also carried out reference calculations with nonpolarizable SPC/E water.^23^

The rate of exchange of the solvation shell water molecules, in the order of milliseconds for Mg^2+^,^24^ which makes such processes difficult to sample by MD techniques. We used Hamiltonian replica exchange molecular dynamics (HREMD) to ensure representative sampling within a feasible simulation time.^25^ The change in the Hamiltonian can be described as

$$U=\left(1-\lambda \right)U^{A}+\lambda U^{B}$$ 3

where U is the total energy. In the “A-state”, σ = 2.6 Å, and in the “B-state”, σ = 3.6 Å, see Table 1. Below, we refer to these Hamiltonians as “small ions” and “large ions”. The former, together with ε = 0.1 kJ/mol, resulted in an ion size close to that for Mg^2+^. For the large ions, water molecules in the cation solvation shell were readily replaced on a time scale of tens of picoseconds. For the small ions, water molecules in the cation solvation shell were not replaced on the time scale of the simulation, 30–50 ns. Only through replica exchanges could the replacement of water and ions in the solvation shell of small ions thus be sampled.

As the acceptance ratio of the replica exchange moves depends on the total energy, the use of this method places limitations on the system size such that a larger system requires a larger number of replicas. The system considered consisted of 12 ion pairs and 665 water molecules, corresponding to an approximately 1 m solution. As this concentration falls within a region characterized by a short screening length, this size, corresponding to a box length of 2.7–2.8 nm, was found to be adequate. Sixty-four replicas were used, with the values of λ chosen with an uneven spacing, such that the acceptance ratio for exchanges was sufficiently removed from zero over the entire range in λ. Trajectories were saved for analysis for λ values of 0.000, 0.168, 0.332, 0.501, 0.664, 0.838, and 1.000.

In addition to full charges on the sulfate ion and metal cation, we also considered a monovalent ion model with the same Lennard-Jones parameters, where all charges are halved. As sulfate and perchlorate are isoelectronic and have the same geometry, this model can be conceptualized as a not-necessarily optimal model for a monovalent metal perchlorate. We also considered a model with an ion charge of 1.5 unit charges, where all charges are reduced to three-quarters of the value in Table 1. The HREMD technique was not required for these reduced-charge models; ordinary MD simulations were performed for the same λ values that were analyzed.

The 1.5-valent model is in some ways similar to an “electrostatic continuum correction” (ECC) model, in which it is assumed that the polarizability is approximately evenly distributed in space and thus can be modeled as a dielectric continuum.^26–28^ Thus, the polarizability in such models globally decreases the strength of electrostatic interactions. Confusingly, this is often represented by scaling down the ionic charges rather than using a relative permittivity greater than one in Coulomb’s law. The scaling factor appropriate for water would yield ionic charges of a magnitude close to 1.5 unit charges for divalent ions. Note, however, that the 1.5-valent models considered here do include explicit polarizabilities that are not typically included in ECC models.

The simulations were carried out in the NPT ensemble at 300 K using the Bussi thermostat and ambient pressure using the analogous rescaling barostat.^29^ Initial configurations were taken from simulations in the λ = 1 state, which itself started from a state with an approximately uniform ion distribution, and equilibrated for 4 ns before data collection started. The typical trajectory length for each replica was tens of nanoseconds by using a 2 fs time step. Due to the replica exchange moves, the simulation time is not to be interpreted as physical time but as a measure of the amount of sampling. The run parameters were chosen such that each replica was able to “diffuse” over the entire range of λ within the simulation time. Long-range electrostatics were treated using the particle-mesh Ewald methods with a real-space cutoff of 8 Å. All MD simulations were carried out using the Gromacs program package, version 2024.^30^ We caution that some earlier versions, e.g., 2021.1, contain errors that render the built-in HREMD code unusable.

## Results and Discussion

The metal cation-sulfate sulfur radial distribution functions for the Mamatkulov model are shown in Figure 2. The difference between the polarizable and nonpolarizable water simulations for divalent ions is dramatic. For the polarizable model with large ions, CIP, corresponding to the closest two peaks, is moderately abundant. For the smaller ions, on the other hand, contact ion pairing is almost completely suppressed, in favor of SIP. The curves for intermediate values of λ show a progressive decrease in the population of CIPs for smaller ions. With nonpolarizable water, the ions are strongly associated, and it appears likely that the salt would form a crystal if the system size allowed this. The small ions represent an exception where the ions tend to form smaller clusters, typically two anions and two cations.

The metal cation—water oxygen radial distribution is shown in Figure 1. The ion size, as quantified by the location of the first peak, varies with λ. The value corresponding to the smaller ions, λ = 0, gives a peak position close to that expected for Mg^2+^.^15^ For the mono and 1.5-valent model variants, the ion sizes are larger, as should be expected due to the fact that the short-range repulsion from the LJ potential does not have to balance as strong an electrostatic attraction.

**Figure 1:**
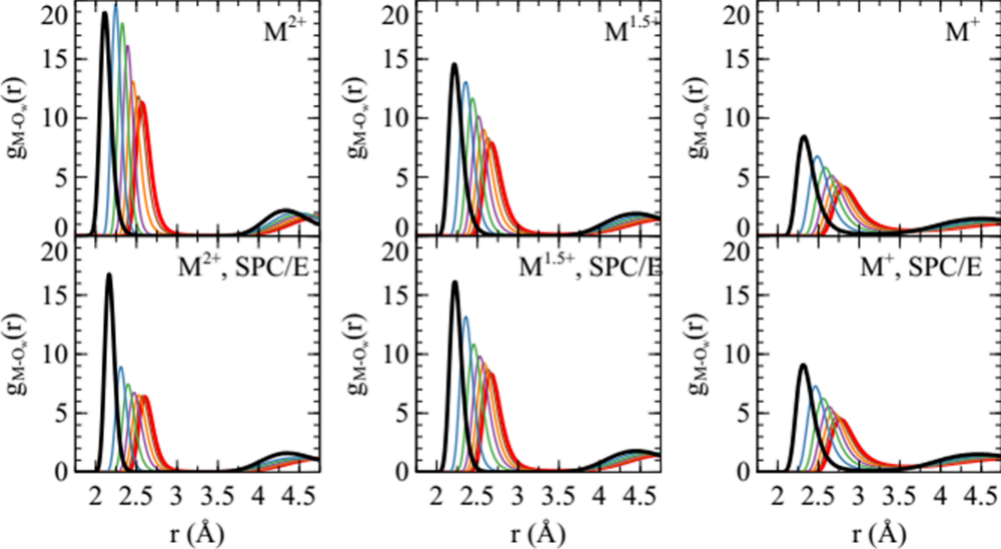
Radial distribution functions between metal cations and water oxygen atoms in the model metal sulfate solutions for models with the ion charge indicated. Black corresponds to λ = 0, red to λ = 1 and the intervening colors blue, green, purple, orange, and brown correspond to λ values of 0.168, 0.332, 0.501, 0.664, and 0.838, respectively. The upper three panels are for simulations with polarizable water (SWM4-NDP model), and the lower three panels are for simulations with nonpolarizble water (SPC/E model).

**Figure 2:**
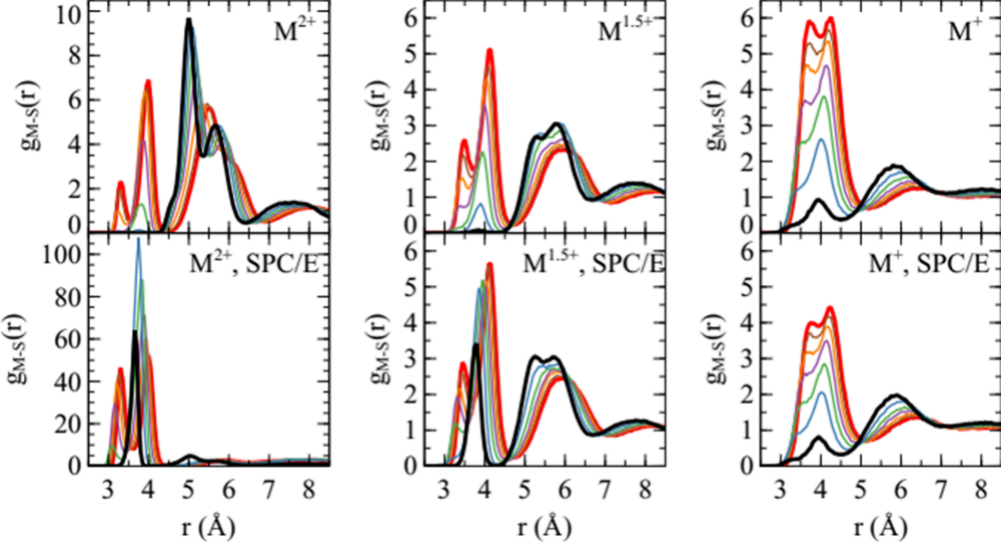
Radial distribution functions between metal cations and the sulfate sulfur atom, for the versions of the Mamatkulov model with the ion charge indicated. Black corresponds to λ = 0, red to λ = 1 and the intervening colors blue, green, purple, orange and brown correspond to λ values of 0.168, 0.332, 0.501, 0.664 and 0.838, respectively. The upper three panels are for simulations with polarizable water (SWM4-NDP model) and the lower three panels are for simulations with nonpolarizble water (SPC/E model).

In the 1.5-valent model variant, both the nonpolarizable and polarizable versions of the model give rise to an unambiguously dissolved state. Except for the population of CIP, which is much smaller for the small cations than for the large cation with the polarizable water model, the two water models give similar solution structures. For the monovalent model variant, there are no qualitative differences between polarizable and nonpolarizable water models. Quantitatively, contact ion pairing is more predominant with the polarizable water model. This observation is interesting in the context of ECC models. While this approach has been successfully applied to correct ‘over association’ with divalent ions,^26^ the present results suggest that this type of model may still overestimate the population of CIP.

For the Cannon sulfate model, the metal cation-sulfate sulfur radial distribution functions are shown in Figure 3. While the quantitative differences compared to the Mamatkulov model are in some cases quite large, the effect of having a polarizable water model is similar. The formation of CIP is suppressed for the divalent and 1.5-valent model compared to the corresponding simulations with nonpolarizable water. This difference increased in all cases for small ions compared to large. This trend is not seen for monovalent ions, for which there is a slightly higher proportion of CIP with polarizable water.

**Figure 3:**
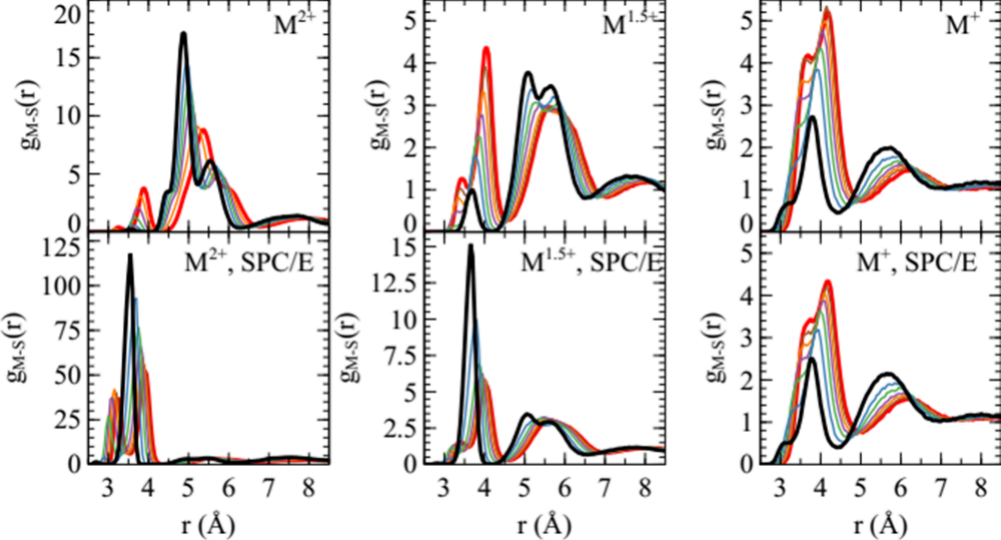
Radial distribution functions between metal cations and the sulfate sulfur atom, for the versions of the Cannon model with the ion charge indicated. Black corresponds to λ = 0, red to λ = 1 and the intervening colors blue, green, purple, orange, and brown correspond to λ values of 0.168, 0.332, 0.501, 0.664, and 0.838, respectively. The upper three panels are for simulations with polarizable water (SWM4-NDP model), and the lower three panels are for simulations with nonpolarizble water (SPC/E model).

For both models, there is a tendency for CIP formation to decrease with increasing ion valence with the polarizable water model and to increase with increasing ion valence for the nonpolarizable water model. A broad-brush explanation for this difference between polarizable and nonpolarizable models can be found by noting that the interaction energy between ions in a charge symmetric electrolyte varies with ion charge *q* as *q* ^2^. As the solvent charges are fixed, the ion–solvent interaction, on the other hand, should vary as *q* if there are no dramatic structural changes of the solvation shell. Thus, the ion–ion interaction will always dominate the ion–solvent interaction for sufficiently large ionic charges. In polarizable models, however, the polarization energy of the first solvation shell will contribute a stabilizing term that varies as *q* ^2^.

This can thus in principle compete with the increase in direct ion–ion interaction; which of the terms will ‘win’ depends on ionic sizes and polarizabilities. The expected *1/r* ^4^ form for the polarization energy term suggests a strong dependence of the population of the CIP on the cation size. We computed the difference in induced dipole moment of water molecules in the first solvation shell of cations, corresponding to a metal–water oxygen distance less than 3.2 Å, and water molecules not in the first solvation shell. This quantity, Δμ_ind_, is shown in Figure 4 as a function of the peak position in the metal ion - water oxygen radial distribution function, *d*_M–Ow_, for the various values of λ.

**Figure 4:**
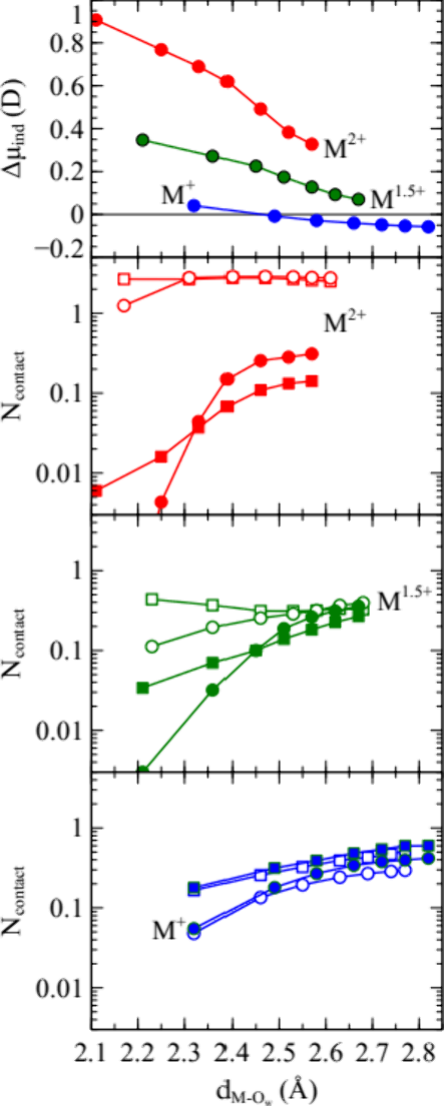
Each point corresponds to a value of λ between 0 and 1. They are ordered according to cation–water distance, *d*_M–Ow_, in the first solvation shell as the connection between the λvalue and ion size is not obvious. Upper panel: excess-induced dipole moment of water molecules in cation solvation shell relative to water molecules not in cation solvation shell, Δμ_ind_, versus *d*_M–Ow_. Lower three panels: population of contact ion pairs, *N*_contact_, versus *d*_M–Ow_. Filled symbols represent polarizable water and empty symbols nonpolarizable water; circles represent the Mamatkulov model, and squares represent the Cannon model.

For the divalent ions, Δμ_ind_ reaches about 1 D for the small ions. As the average induced dipole moment in bulk SWM4-NDP water was found to be 0.65 D, this effect must be considered large. Even for the large ions, Δμ_ind_ is significantly greater than zero. For the 1.5-valent model variant, Δμ_ind_ is positive for all values of lambda but approaches zero for large ions. In contrast, Δμ_ind_ is close to zero for the monovalent model variant and negative for the large ions and for most values of λ. The population of CIP, *N*_contact_, computed as the integral of the cation–sulfate S radial distribution function up to the minimum that separates the CIP from the SIP peaks, is shown in the lower three panels of Figure 4. This definition allows anions to simultaneously “pair” with more than one cation and consequently include triple ions and larger aggregates; values of *N*_contact_ greater than one imply that larger aggregates are necessarily formed. For the divalent cations, the formation of CIP is dramatically smaller with the polarizable water model. The same trend can be seen for the 1.5-valent model, while the trend is weaker and in the opposite direction for the monovalent model variant.

This provides an illustration of the seemingly disproportionate importance of water polarization for multivalent ions. As the polarization of the first solvation shell of the monovalent cations is similar to that of bulk water, solvent shell polarization has only a minor effect for such ions. As it is not the valency of the ion itself but the electric field strength felt by the solvation shell water molecules that is decisive, small monovalent ions such as Li^+^ can be expected to show a stronger-than-bulk polarization of the first solvation shell. Conversely, larger multivalent ions do not necessarily have a strongly polarized solvation shell. For sulfate, we found deviations from the bulk polarization, in line with a previous report.^18^ However, these were on the order of 0.1 D, significantly smaller than the polarization of the cation solvation shells.

Several sulfates with divalent cations are highly soluble and the osmotic coefficients of the sulfates of Be^2+^, Mg^2+^, Cu^2+^, Zn^2+^, Ni^2+^, Cd^2+^, and Mn^2+^ are known.^31^ This is of special interest in the present context as the osmotic properties of salt solutions depend sensitively on the solvent-averaged interaction between ions. A larger osmotic coefficient for a given concentration indicates that repulsive forces are more prominent. As can be seen in Figure 5, beryllium sulfate gives the largest osmotic coefficients over almost the whole concentration range, even though Be^2+^ is the smallest cation by a wide margin. We have previously observed the tendency that an extremely small ion behaves as if it was extremely large in that lithium gives a larger effective radius than larger alkali metal ions when the radii are selected to reproduce the activity coefficients of the alkali metal halides in the primitive model (PM),^32^ where solute ions are modeled as a plasma of hard spheres and the water solvent is only implicitly represented through the choice of model parameters. Below, we perform a similar analysis for the set of divalent metal sulfates.

**Figure 5:**
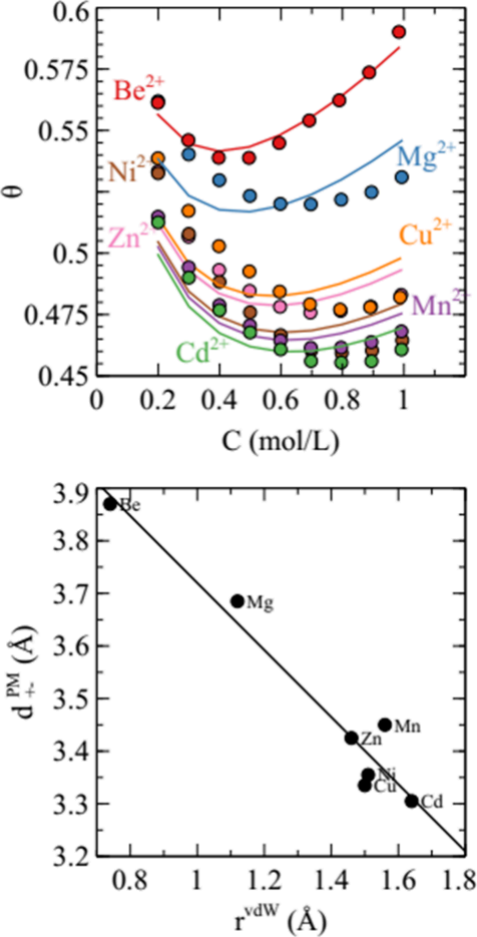
Upper panel: experimental osmotic coefficients from ref 31 for the sulfate of the metal indicated (symbols) and the osmotic coefficient from the PM with the best-fit cation radius (lines). Lower panel: anion–cation distance of closest approach for the best-fit cation diameters plotted against the ion van der Waals radius as estimated in ref 35.

The PM is defined by the pair interaction potential

$$U_{ij}^{\mathrm{PM}}(r)=U_{ij}^{\mathrm{Coul}}(r)+U_{ij}^{\mathrm{core}}(r)$$ 4

where *U*_*ij*_^Coul^(*r*) is the Coulomb potential given by

$$U_{ij}^{\mathrm{Coul}}(r)=\frac{q_{i}q_{j}}{4\pi \epsilon \epsilon_{0}r}$$ 5

where *q*_*l*_, *l = i, j*, is the ionic charge, ϵ_0_ is the permittivity of vacuum, and ϵ is the relative permittivity of the solution, the value of which is taken as that of pure water, 78.36 at 25 °C.^33^ The term *U*_*ij*_^core^(*r*) is a hard sphere potential that is zero for *r* ≥ *d*_*ij*_ and infinite for *r* < *d*_*ij*_, where *r* is the distance between the ions and *d*_*ij*_ is their distance of closest approach, equal to the hard sphere diameter for pairs of like ions. A larger distance of closest approach corresponds to a greater predominance of repulsive interactions, and we expect a good fit to the data only if the interionic potential of mean force is monotonically and steeply repulsive. We assume that the ionic radii are additive, as is the case if the hard sphere model is taken at face value, so that *d*_+–_ = (*d*_++_ + *d*_––_)/2. We expect that *d*_+–_ is the most important model parameter, as it determines the strength of the Coulomb interaction at cation–anion contact and thereby the degree of ion pairing. Here, we keep *d*_––_ fixed to 4.6 Å,^34^ and use *d*_++_ as a fitting parameter; *d*_+–_ follows from additivity. We computed the PM osmotic coefficient using the hypernetted chain approximation in the fitting process and tested the final model parameters using Monte Carlo simulations; see the SI.

As can be seen in Figure 5, the PM gives a good fit to the osmotic coefficient data only for beryllium sulfate, which is the smallest ion in the set. For all other cations, the minimum in the curve occurs at very low concentrations. The optimum value for *d*_+–_ appears inversely correlated with the cation van der Waals radius,^35^ at least in the sense that the two smallest ions Mg^2+^ and Be^2+^ behave as anomalously large. For the five larger ions, the van der Waals radius and *d*_+–_ both vary in a narrow range, and it is unclear if there is a meaningful relationship between the properties for this subset. This is qualitatively in line with the observation that ion pairing is totally suppressed only below a certain ion size, and the ion pairing behavior of larger ions is determined by a number of factors.

The best-fit radius for Mg^2+^ in magnesium halides obtained in ref 32 was significantly larger than the value obtained here for magnesium sulfate. The current comparison therefore does not suggest that the cation radii obtained for electrolytes of the 2:1 valence type are transferable to 2:2 electrolytes. In contrast to the situation in magnesium halides, the cation radii obtained here are too small to be interpreted to correspond to cations that are always surrounded by solvation water molecules. The fact that the trend in osmotic coefficient with concentration is reproduced only for beryllium sulfate suggests that only for the tiny Be^2+^ ion is the solvation shell so strongly bound that the solvated ion actually resembles a hard sphere.

Models that lack explicit polarization, including both traditional nonpolarizable force fields and ECC models, can be viewed as solvent-averaged models with respect to this property. The dramatic modification of the Mg^2+^ sulfate O interaction in ref 11 was presented in that work as an *ad hoc* measure to correctly reproduce the experimental activity derivative, similar to the procedure used to fit *d*_+–_ for the PM. The results presented here provide a mechanistic explanation for why such a modification was necessary. We expect that a similar comparison to dielectric spectra as in ref^11^ would yield a similar conclusion that the dominance of SIP in MgSO_4_ is in line with the experiments, but we cannot directly carry out such an analysis on our trajectories, as the HREMD technique does not have long-time dynamics. Careful tuning of the interaction between individual particle pairs, for instance, by NBFIX in the CHARMM force field, has been presented as an alternative approach to correct for “overbinding” by cations. The results presented above suggest that both of these are needed in the special case of sulfates with divalent cations. While it is convenient to directly modify pair interactions in cases where just one or a few of them are decisive, it is less so if all or a majority of the pair interactions require individual attention.

The circumstance that water molecules in the solvation shells of monovalent ions have about the same induced dipole moment as in bulk water appears fortuitous but has far-reaching implications. It may well be that it is a necessary condition for nonpolarizable models to be acceptable. The failure of the conventional charged-LJ-particle model of ions to simultaneously reproduce the solvation energy and ion–water distance for divalent ions in ref 15 may well be the norm, and the relative success for monovalent ions the exception. The fact that polarizable models accurately reproduce the predominance of SIP over CIP in sulfates with small metal cations can likely be explained on the basis of the same physical insight. It is not necessarily the case, though, that a pairwise additive *1/r* ^4^ interaction potential will be able to quantitatively reproduce the effect. A water molecule that participates in an SIP is exposed to the vector sum of fields from the cation and ion, and it is the square of the magnitude of this field that enters eq 1. Thus, solvent shell polarization is likely to have an even greater effect in the context of ion pairing than in the description of the solvation shells of individual ions.

The approach in ref 15 to include a corrections term for the polarization energy in an otherwise nonpolarizable model can be generalized to not just include the cation–water interaction but all species. While the polarization energy given by eq 1 is not a pairwise additive, it depends only on the electric field, which is additive as a vector sum. In principle, an approximation of the polarization energy where each particle is polarized by the field from just the permanent charges can be included in the Hamiltonian. This corresponds to the recognition that even in situations where the fluctuations in induced dipole moment are not significant enough to consider, the energetic consequences of polarization may need to be accounted for. This is not a new insight, but the one that gave rise to the still-widely used SPC/E water model.^23^ This approach bypasses the computationally demanding and possibly unstable calculation of the self-consistent induced dipole moments that constitute the main added difficulty of using a fully polarizable model.

## Conclusions

MD simulations with replica exchange methodology were performed to explore the effect of the polarizability of solvating water molecules of divalent cations in metal sulfates on the formation of different types of ion pairs. Small divalent cations such as Mg^2+^ highly polarize the solvating water molecules, which results in the absence of contact ion pairs but the presence of solvent-separated ion pairs in the solution. On the other hand, large cations like Zn^2+^ weakly polarize the solvating water molecules, which leads to the formation of contact ion pairs. MD simulations with a nonpolarizable water model dramatically overestimated the presence of contact ion pairs, thus highlighting the importance of correctly taking into account the polarizing effects in the atomistic simulations of salt solutions.

## Supplementary Material



## Notes

Details of the PM calculations (PDF)
