# Supplementary material for: Ion Pairing and Solvent Shell Polarization in Aqueous Solutions of Divalent Metal Sulfates
Source: J Phys Chem B. 2025 Aug 6;129(33):8541–7. doi: 10.1021/acs.jpcb.5c03061 (PMC12376091; doi:10.1021/acs.jpcb.5c03061)
Supplement: Supplementary file 1 [file jp5c03061_si_001.pdf]

Supporting information for 'Ion pairing and solvent shell polarization in aqueous solutions of divalent metal sulfates'

*Erik Bialik and Zareen Abbas**

Department of Chemistry, University of Gothenburg SE-413 90 Gothenburg, Sweden

Fitting of effective ion sizes to experimental data

Experimental activity coefficients and osmotic coefficients of sulfates with the cations Be^{2+}, Mg^{2+}, Mn^{2+}, Ni^{2+}, Cu^{2+}, Zn^{2+} and Cd^{2+} were taken from ref. 1. To cast the osmotic coefficient in a form that is compatible with the definition of this quantity in McMillan- Mayer type models, based on number density, the data were converted from Lewis-Randall to McMillan-Mayer scale according to the procedure described in.[2] Empirical expressions for the densities of the salt solutions from ref. 3 were used.

The activity coefficient data are based on isopiestic measurements of the osmotic co- efficient for concentrations above some lowest concentration m_0, 0.1 mol/kg in this case, followed by integration according to the Gibbs-Duhem equation,

$$-ln\big(\gamma(m)\big) = -ln\big(\gamma(m_0)\big) + \int_{m_0}^{m}\big(1-\phi(m')\big)dm' \quad (1)$$

The osmotic coefficients and activity coefficients thus contain essentially the same information. The accuracy of the absolute value of the activity coefficient is dependent on the activity coefficient for concentration m_0. If $\gamma(m_0)$ is not reliably known, only the *ratio* of activity coefficients (or equivalently, difference in excess chemical potential) to that for a given concentration can be calculated. For this reason, the osmotic coefficients were used to determine the optimal parameters for each model. In ref. 1, $\gamma(m_0)$ is simply set to 0.150 (on the Lewis-Randall scale, though the difference between the McMillan-Mayer and Lewis-Randall scale are insignificant for this concentration) for all the divalent sulfates. (See ref. 4 for details.)

In order to obtain meaningful information about the ionic interactions from such a fit the number of parameters must be kept to a minimum. Otherwise, there is a risk that the model becomes so flexible that a good fit can be obtained regardless of any resemblance between the model potential and the true potential. What number of parameters is reasonable ultimately depends on the number of independent pieces of information that has to be simultaneously fitted. Because we limit ourselves to a single type of information here, we consider it prudent to use a single fitting parameter, d_{++}

The root mean square deviation between the experimental and theoretical osmotic coefficients,

$$\delta = \left[\frac{\sum_i^N \left(\phi(n_i)-\phi^{exp}(n_i)\right)^2}{N}\right]^{1/2} \qquad (2)$$

where $\varphi(n_i)$ and $\varphi^{exp}(n_i)$ are the theoretical (from the HNC approximation) and experimental osmotic coefficient for concentration n_i and N is the number of different concentrations considered, was used as the target quantity for the fit. We have used the HNC approximation in order to calculate the model osmotic coefficient because the accurate evaluation of osmotic coefficients

from simulations would require very long runs. The value of the fitting parameter that minimized δ was considered best. The results thus obtained are subsequently validated by comparison of the activity coefficients to the results from simulations for the optimal parameters. The nine data point corresponding to 0.2 to 1.0 m concentration was used, i.e. $N = 9$. It is not reasonable to expect McMillan-Mayer type models to be applicable for concentrations much higher than 1 or 2 M, although the limit of the range of validity cannot be known *a priori*. The lower limit of 0.2 m is imposed due to the fact that the HNC approximation is not accurate for the PM at low concentrations under conditions of high electrostatic coupling. Solutions of different divalent sulfates have different density. Thus the osmotic coefficients are not calculated at exactly the same concentration interval in terms of molar concentrations. However, the difference between the salts in the width of the concentration interval is small, less than one percent, and not likely to skew the comparison between the fits for different cations.

For all divalent sulfates considered here the cation diameter was adjusted to the value that minimized δ while the anion diameter was fixed to 4.6 Å, the crystallographic diameter of a sulfate ion. The results are summarized in the main text. The optimal values of δ are in the order of one to a few percent of the typical values of the osmotic coefficients in the concentration range considered.

The HNC approximation

Whenever no great error is incurred by doing so, it is preferable to use integral equation theory over simulation, as the former requires much less computational effort, for evaluating the properties of a given model. The HNC approximation have been tested before,[5] showing great promise as a

quantitative theory *in some regions of parameter space*, though not in others.[6] For this reason, the accuracy of the HNC approximation must be tested against simulation whenever the interaction potential considered deviates from those for which the HNC approximation has previously been tested.

The Ornstein-Zernike (OZ) equation for a bulk system is given by

$$h_{ij}(r) = c_{ij}(r) + \sum_k n_k \int h_{ik}\left(|r - r'|\right) c_{kj}(r') dr' \quad (3)$$

where $h_{ij}(r)$ is the total correlation function and $c_{ij}(r)$ is the direct correlation function for ion species i and j. The total correlation function is related to the pair distribution function, $g_{ij}(r)$, by $g_{ij}(r) = h_{ij}(r) + 1$. The hypernetted chain (HNC) approximation has the form of a relation between $h_{ij}(r)$ and $c_{ij}(r)$,

$$c_{ij}(r) = -\beta u_{ij}(r) + h_{ij}(r) - ln\left[1 + h_{ij}(r)\right] \quad (4)$$

Together, these two relations between $h_{ij}(r)$ and $c_{ij}(r)$ form a system of equations from which these functions can be calculated given $u_{ij}(r)$. Here, the standard procedure of solving the set of equations composed of (3) and (4) by Picard iteration is employed.[7] The numerical difficulties caused by the long-range Coulomb interactions are handled by the method described in ref. 8. Briefly, for a starting guess for the set of c_{ij} eq. (3) is solved with respect to the set of h_{ij} by taking the Fourier transform of this equation. Due to the convolution theorem eq. (3) then takes the form of an algebraic equation that is solved for the Fourier transform of the set of h_{ij}. Then the inverse Fourier transforms are calculated and a new guess for the set of c_{ij} is obtained from eq. (4). The procedure is repeated until convergence.

The accuracy of the HNC approximation tends to be good for ionic systems. A known exception is systems with strong, attractive electrostatic interactions at low density. For PM electrolytes there is a region where there is no solution for the HNC approximation. This region has been mapped out for the "restricted primitive model", i.e. the PM with the constraints $d_{++} = d_{--} = d_{+-}$ and $Z_{+} = -Z_{-}$.[9] In the vicinity of the region of no solution there are systematic errors in the total correlation functions. For the anion-cation correlation function the height of the peak at contact is then too low; the degree of ion pairing in the system is underestimated. In the like-charged ion correlation functions the errors of the HNC approximations show up as a spurious peak about one ion radius from the contact distance. [10] At higher concentrations both these errors are less important: the peak height in the unlike- charged ion correlation function is better represented and the peak in the like-charged ion correlation function is here a true feature of the system. The error in the HNC approximation is that this peak does not disappear for low concentrations. For aqueous 2:2 electrolytes with sizes around those considered here, the region where this problem becomes severe starts at concentrations below about 0.2 M and rapidly gets worse for lower concentrations. Moreover, the HNC approximation also shows a more severe inconsistency between the compressibility and virial routes for calculation of thermodynamic properties for 2:2 salts than for 1:1 and 1:2 salts. The accuracy of the HNC approximation thus cannot be taken for granted in the present case. For this reason, MC simulations are employed to test the predictions about the activity coefficients from HNC theory. Note that the HNC approximation is thermodynamically consistent in the limited sense that the "energy" route to thermodynamic properties is consistent with the "virial" route.[11,12] Because of the presence of a region where no solution exists the compressibility route is unpractical for the

calculation of osmotic coefficients and activity coefficients on an absolute scale for the PM of solutions of divalent sulfates. For this reason all thermodynamic quantities presented in this work are calculated via the energy/virial route.

MC simulations

All MC simulations presented in this work are performed using the standard Metropolis Monte Carlo method for the canonical ensemble using 242 pairs of ions with the cubic box size determined by the concentration. The typical dimension is 202 Å of box length. Periodic boundary conditions with the minimum image convention, which guarantees that the energy is calculated for an electroneutral set of ions, were used. The ions were initially placed randomly, discarding configurations with hard-sphere overlaps, and the simulation was run for 50 million configurations of equilibration followed by 150 million configurations. The long equilibration was performed in order to guard against the possibility that non-representative configurations formed by the initial random placement of the particles would persist for many steps due to the strong forces acting in 2:2 salts.

The excess chemical potential was evaluated using a variant of the Widom insertion method. The basic implementation of this method is that a trial insertion of a particle is made at random into the simulation box and the interaction with all other particles is evaluated. The excess free energy in units of $k_B T$ is simply the ensemble average of the Boltzmann factor

associated with the insertion of a particle at a random location. In charged systems, the naive implementation of this method displays very poor convergence with increasing system size. This is because the insertion gives rise to a non-electroneutral system. To remedy this problem an approximate but accurate modification of this scheme was used.[13]

REFERENCES

1. Robinson, R. A.; Stokes, R. H. *Electrolyte Solutions*; Dover Publications, 2002.
2. Pailthorpe, B. A.; Mitchell, D. J.; Ninham, B. W. *J. Chem. Soc. Faraday Trans.* **1984**, *80*, 115.
3. Novotný, P.; Söhnel, O. *J. Chem. Eng. Data* **1988**, *33*, 49.
4. Robinson, R. A.; Stokes, R. H. *Trans. Faraday Soc.* **1949**, *45*, 612.
5. Valleau, J. P.; Cohen, L. K.; Card, D. N. *J. Chem. Phys.* **1980**, *72*, 5924.
6. Duh, D.-M.; Haymet, A. D. J. *J. Chem. Phys.* **1992**, *97*, 7716.
7. Rasaiah, J. C.; Friedman, H. L. *J. Chem. Phys.* **1968**, *48*, 2742.
8. Ng, K.-C. *J. Chem. Phys.* **1974**, *61*, 2680.
9. Belloni, L. *J. Chem. Phys.* **1993**, *98*, 8080.

10. Rossky, P. J.; Dudowicz, J. B.; Tembe, B. L.; Friedman, H. L. *J. Chem. Phys.* **1980**, *73*, 3372.

11. Morita, T. *Prog. Theor. Phys.* **1960**, *23*, 829.

12. Schlijper, A. G.; da Gama, M. M. T.; Ferreira, P. G. *J. Chem. Phys.* **1993**, *98*, 1534.

13. Svensson, B. R.; Woodward, C. E. *Mol. Phys.* **1988**, *64*, 247.
